# Supplementary material for: Sustained fitness gains and variability in fitness trajectories in the long-term evolution experiment with Escherichia coli
Source: Proc Biol Sci. 2015 Dec 22;282(1821):20152292. doi: 10.1098/rspb.2015.2292 (PMC4707762; doi:10.1098/rspb.2015.2292)
Supplement: Supplementary Materials [file rspb20152292supp1.pdf]

Sustained fitness gains and variability in fitness trajectories in the long-term evolution experiment with *Escherichia coli*

Richard E. Lenski, Michael J. Wiser, Noah Ribeck, Zachary D. Blount, Joshua R. Nahum, J. Jeffrey Morris, Luis Zaman, Caroline B. Turner, Brian D. Wade, Rohan Maddamsetti, Alita R. Burmeister, Elizabeth J. Baird, Jay Bundy, Nkrumah A. Grant, Kyle J. Card, Maia Rowles, Kiyana Weatherspoon, Spiridon E. Papoulis, Rachel Sullivan, Colleen Clark, Joseph S. Mulka, Neerja Hajela

SUPPLEMENTARY MATERIALS

Table S1. The *E. coli* population samples and strains used in this study.

ID Number	Description
REL10999	Whole-population sample from Ara+1 at generation 40,000
REL10933	Whole-population sample from Ara+2 at generation 40,000
REL10934	Whole-population sample from Ara+3 at generation 40,000
REL10935	Whole-population sample from Ara+4 at generation 40,000
REL10977	Whole-population sample from Ara+5 at generation 40,000
REL10926	Whole-population sample from Ara–1 at generation 40,000
REL10929	Whole-population sample from Ara–4 at generation 40,000
REL10930	Whole-population sample from Ara–5 at generation 40,000
REL10998	Whole-population sample from Ara–6 at generation 40,000
REL11383	Whole-population sample from Ara+1 at generation 50,000
REL11325	Whole-population sample from Ara+2 at generation 50,000
REL11326	Whole-population sample from Ara+3 at generation 50,000
REL11327	Whole-population sample from Ara+4 at generation 50,000
REL11362	Whole-population sample from Ara+5 at generation 50,000
REL11318	Whole-population sample from Ara–1 at generation 50,000
REL11321	Whole-population sample from Ara–4 at generation 50,000
REL11322	Whole-population sample from Ara–5 at generation 50,000
REL11382	Whole-population sample from Ara–6 at generation 50,000
REL11765	Whole-population sample from Ara+1 at generation 60,000
REL11686	Whole-population sample from Ara+2 at generation 60,000
REL11687	Whole-population sample from Ara+3 at generation 60,000
REL11688	Whole-population sample from Ara+4 at generation 60,000
REL11723	Whole-population sample from Ara+5 at generation 60,000
REL11678	Whole-population sample from Ara–1 at generation 60,000
REL11681	Whole-population sample from Ara–4 at generation 60,000
REL11682	Whole-population sample from Ara–5 at generation 60,000
REL11763	Whole-population sample from Ara–6 at generation 60,000
REL10948	Clone A sampled from Ara–5 at generation 40,000
REL11638	Spontaneous Ara^+ mutant derived from REL10948
REL606	Ancestral strain for the long-term evolution experiment
REL11709	Clone A sampled from Ara+4 at generation 60,000
REL11710	Clone B sampled from Ara+4 at generation 60,000
REL11711	Clone C sampled from Ara+4 at generation 60,000
REL11728	Clone A sampled from Ara+5 at generation 60,000
REL11729	Clone B sampled from Ara+5 at generation 60,000
REL11730	Clone C sampled from Ara+5 at generation 60,000

Table S2 Estimated mean fitness of nine LTEE populations at three time points, measured relative to a common competitor isolated from one population at 40,000 generations.

	40,000 Generations			50,000 Generations			60,000 Generations		
**Population**	**N**	**Mean**	**SD**	**N**	**Mean**	**SD**	**N**	**Mean**	**SD**
**Ara+1**	41	0.9467	0.0369	39	0.9682	0.0342	42	0.9708	0.0202
**Ara+2**	41	1.0253	0.0288	42	1.0388	0.0581	39	1.0739	0.0525
**Ara+3**	41	1.0933	0.0367	42	1.1251	0.0387	39	1.1449	0.0500
**Ara+4**	42	0.9999	0.0264	42	1.0079	0.0266	41	1.0598	0.0366
**Ara+5**	41	0.9870	0.0281	41	1.0164	0.0249	42	1.0422	0.0372
**Ara−1**	42	1.0387	0.0471	42	1.0846	0.0604	41	1.1145	0.0592
**Ara−4**	41	1.0584	0.0341	41	1.0661	0.0510	41	1.1058	0.0377
**Ara−5**	41	0.9909	0.0204	42	1.0256	0.0221	41	1.0447	0.0261
**Ara−6**	42	1.0217	0.0194	42	1.0383	0.0262	42	1.0588	0.0215
**Grand mean**	9	1.0180	0.0432	9	1.0412	0.0459	9	1.0684	0.0504

Table S3. ANOVAs testing among-population variation in fitness at 40,000 generations.

All nine populations

Source	SS	df	MS	*F*	*p*	Friedman *p*
**Population**	0.58552	8	0.07319	89.618	<0.0001	<0.0001
**Block**	0.09356	36	0.00260	3.182	<0.0001	
**Error**	0.23521	288	0.00082			
**Total**	0.91429	332	0.00275			

Excluding three hypermutator populations

Source	SS	df	MS	*F*	*p*	Friedman *p*
**Population**	0.16364	5	0.03273	52.091	<0.0001	<0.0001
**Block**	0.04599	36	0.00128	2.033	<0.0001	
**Error**	0.11310	180	0.00063			
**Total**	0.32273	221	0.00146			

Also excluding population Ara+1

Source	SS	df	MS	*F*	*p*	Friedman *p*
**Population**	0.05289	4	0.01322	23.899	<0.0001	<0.0001
**Block**	0.03058	36	0.00085	1.535	0.0408	
**Error**	0.07966	144	0.00055			
**Total**	0.16313	184	0.00089			

Table S4. ANOVAs testing among-population variation in fitness at 50,000 generations.

All nine populations

Source	SS	df	MS	*F*	*p*	Friedman *p*
**Population**	0.60393	8	0.07549	58.312	<0.0001	<0.0001
**Block**	0.11506	36	0.00320	2.469	<0.0001	
**Error**	0.37284	288	0.00129			
**Total**	1.09183	332	0.00329			

Excluding three hypermutator populations

Source	SS	df	MS	*F*	*p*	Friedman *p*
**Population**	0.13308	5	0.02662	27.859	<0.0001	<0.0001
**Block**	0.05954	36	0.00165	1.731	0.0106	
**Error**	0.17196	180	0.00096			
**Total**	0.36458	221	0.00165			

Also excluding population Ara+1

Source	SS	df	MS	*F*	*p*	Friedman *p*
**Population**	0.03505	4	0.00876	8.796	<0.0001	<0.0001
**Block**	0.04746	36	0.00132	1.323	0.1269	
**Error**	0.14346	144	0.00100			
**Total**	0.22597	184	0.00123			

Table S5. ANOVAs testing among-population variation in fitness at 60,000 generations.

All nine populations

**Source**	**SS**	**df**	**MS**	***F***	***p***	**Friedman *p***
**Population**	0.69848	8	0.08731	82.124	<0.0001	<0.0001
**Block**	0.09046	32	0.00283	2.659	<0.0001	
**Error**	0.27217	256	0.00106			
**Total**	1.06111	296	0.00358			

Excluding three hypermutator populations

**Source**	**SS**	**df**	**MS**	***F***	***p***	**Friedman *p***
**Population**	0.24458	5	0.04892	57.497	<0.0001	<0.0001
**Block**	0.04233	32	0.00132	1.555	0.0405	
**Error**	0.13612	160	0.00085			
**Total**	0.42303	197	0.00215			

Also excluding population Ara+1

**Source**	**SS**	**df**	**MS**	***F***	***p***	**Friedman *p***
**Population**	0.02775	4	0.00694	7.518	<0.0001	<0.0001
**Block**	0.04540	32	0.00142	1.537	0.0491	
**Error**	0.11812	128	0.00092			
**Total**	0.19127	164	0.00117			

Table S6. ANOVAs testing among-population variation in fitness gains between 40,000 and 60,000 generations.

All nine populations

**Source**	**SS**	**df**	**MS**	***F***	***p***	**Friedman *p***
**Population**	0.05047	8	0.00631	4.353	0.0001	<0.0001
**Block**	0.05595	28	0.00200	1.379	0.1055	
**Error**	0.32460	224	0.00145			
**Total**	0.43102	260	0.00166			

Excluding three hypermutator populations

**Source**	**SS**	**df**	**MS**	***F***	***p***	**Friedman *p***
**Population**	0.04233	5	0.00847	7.277	<0.0001	<0.0001
**Block**	0.02563	28	0.00092	0.787	0.7667	
**Error**	0.16286	140	0.00116			
**Total**	0.23082	173	0.00133			

Table S7. ANOVAs testing among-population variation in the deceleration of fitness changes between 40,000 to 50,000 and 50,000 to 60,000 generations.

All nine populations

Source	SS	df	MS	*F*	*p*	Friedman *p*
**Population**	0.16377	8	0.02047	3.974	0.0002	0.0001
**Block**	0.31121	28	0.01111	2.158	0.0011	
**Error**	1.15384	224	0.00515			
**Total**	1.62882	260	0.00626			

Excluding three hypermutator populations

Source	SS	df	MS	*F*	*p*	Friedman *p*
**Population**	0.09176	5	0.01835	3.826	0.0028	0.0012
**Block**	0.19183	28	0.00685	1.428	0.0924	
**Error**	0.67146	140	0.00480			
**Total**	0.95505	173	0.00552			

Table S8. Fluctuation tests to estimate mutation rates using the p_0 method.

Mutations to rifampicin resistance

Strain	Population / Generation	Average Cells Tested	Replicates	Mutation Events	Estimated Mutation Rate
REL606	Ancestor / 0	9.41×10^7	24	6	3.06×10^{-9}
REL11709	Ara+4 / 60,000	2.92×10^7	24	1	1.46×10^{-9}
REL11710	Ara+4 / 60,000	2.32×10^7	24	1	1.84×10^{-9}
REL11711	Ara+4 / 60,000	2.56×10^7	24	0.5	8.24×10^{-10}
REL11728	Ara+5 / 60,000	3.16×10^7	24	1	1.35×10^{-9}
REL11729	Ara+5 / 60,000	5.37×10^7	24	2	1.62×10^{-9}
REL11730	Ara+5 / 60,000	5.10×10^7	24	2	1.71×10^{-9}

Mutations to nalidixic-acid resistance

Strain	Population / Generation	Average Cells Tested	Replicates	Mutation Events	Estimated Mutation Rate
REL606	Ancestor / 0	9.41×10^7	24	1	4.52×10^{-10}
REL11709	Ara+4 / 60,000	2.92×10^7	24	3	4.57×10^{-9}
REL11710	Ara+4 / 60,000	2.32×10^7	24	1	1.84×10^{-9}
REL11711	Ara+4 / 60,000	2.56×10^7	24	0.5	8.24×10^{-10}
REL11728	Ara+5 / 60,000	3.16×10^7	24	0.5	6.66×10^{-10}
REL11729	Ara+5 / 60,000	5.37×10^7	24	0.5	3.92×10^{-10}
REL11730	Ara+5 / 60,000	5.10×10^7	24	0.5	4.13×10^{-10}
